# DeepCoVDR: deep transfer learning with graph transformer and cross-attention for predicting COVID-19 drug response

**DOI:** 10.1093/bioinformatics/btad244

**Published:** 2023-06-30

**Authors:** Zhijian Huang, Pan Zhang, Lei Deng

**Affiliations:** School of Computer Science and Engineering, Central South University, Changsha 410083, China; Hunan Provincial Key Laboratory of Clinical Epidemiology, Xiangya School of Public Health, Central South University, Changsha 410083, China; School of Computer Science and Engineering, Central South University, Changsha 410083, China

## Abstract

**Motivation:**

The coronavirus disease 2019 (COVID-19) remains a global public health emergency. Although people, especially those with underlying health conditions, could benefit from several approved COVID-19 therapeutics, the development of effective antiviral COVID-19 drugs is still a very urgent problem. Accurate and robust drug response prediction to a new chemical compound is critical for discovering safe and effective COVID-19 therapeutics.

**Results:**

In this study, we propose DeepCoVDR, a novel COVID-19 drug response prediction method based on deep transfer learning with graph transformer and cross-attention. First, we adopt a graph transformer and feed-forward neural network to mine the drug and cell line information. Then, we use a cross-attention module that calculates the interaction between the drug and cell line. After that, DeepCoVDR combines drug and cell line representation and their interaction features to predict drug response. To solve the problem of SARS-CoV-2 data scarcity, we apply transfer learning and use the SARS-CoV-2 dataset to fine-tune the model pretrained on the cancer dataset. The experiments of regression and classification show that DeepCoVDR outperforms baseline methods. We also evaluate DeepCoVDR on the cancer dataset, and the results indicate that our approach has high performance compared with other state-of-the-art methods. Moreover, we use DeepCoVDR to predict COVID-19 drugs from FDA-approved drugs and demonstrate the effectiveness of DeepCoVDR in identifying novel COVID-19 drugs.

**Availability and implementation:**

https://github.com/Hhhzj-7/DeepCoVDR.

## 1 Introduction

With the outbreak of COVID-19, the lives and health of people worldwide have been seriously threatened. The pathogen causing the disease has been named SARS-CoV-2 (Guan et al. 2020), and it is the most pathogenic human coronavirus ever discovered (Cha et al. 2018). In order to prevent COVID-19 from further causing people’s health crisis, it is urgent to find SARS-CoV-2 antiviral drugs. Several drugs and vaccines have been approved, but there is still a lack of specific therapeutics to block severe illness and mortality due to the emergence of some new SARS-CoV-2 strains (Spinner et al. 2020).

Recently, certain drugs targeting the viral protein SARS-CoV-2, such as remdesivir (Spinner et al. 2020; Yin et al. 2020), paxlovid (Owen et al. 2021; Hammond et al. 2022), and molnupiravir (Wang et al. 2021b; Jayk Bernal et al. 2022), have been approved by the FDA, but their clinical efficacy remains controversial. Therefore, targeting SARS-CoV-2 host receptors is emerging as another potential solution in the search for specific COVID-19 drugs, in addition to the two entry receptors ACE2 and TMPRSS2, Gordon et al. (2020) identified 332 host–virus protein interactions between SARS-CoV-2 and human proteins through affinity purification mass spectrometry. Moreover, transcriptome profiling also provides an opportunity to predict antiviral drug response by detecting quantitative changes in host gene activity and gene regulation, and changes in the level of gene expression always contribute to altered pathways, which play a crucial role in determining the phenotypes of cells (Sharifi-Noghabi et al. 2019; Riva et al. 2020). Sometimes similar phenotypes can be observed in different cells due to changes in the same gene activity; For example, recent studies have shown increased expression of immune checkpoint receptors, including PD-1 and CTLA-4, in lung tissue from COVID-19 patients, both of which are associated with cancer development (Sharif-Askari et al. 2021). The research on COVID-19 drug prediction mainly focuses on drug repurposing. Traditional drug repurposing methods rely on expensive and time-consuming wet-lab experiments (Avorn 2015), and computational methods in drug repurposing can effectively alleviate these problems.

So far, some computational drug repurposing methods for screening SARS-CoV-2 antiviral drugs have been proposed, which can be mainly divided into two categories. The first category is the network-driven method, which analyzes the information extracted from the similarity of drugs and other nodes (such as viruses, proteins, etc.). Zhou et al. (2020) presented an integrative antiviral drug repurposing methodology that searches for potential SARS-CoV-2 antiviral drugs by computing the network proximity among the drug’s target proteins and HCoV-related proteins. Meng et al. (2021) proposed the SCPMF method which integrated the known drug–virus interaction, drug–drug similarity, and virus–virus similarity network to construct a heterogeneous network, and used the similarity constraint probability matrix decomposition method to search for COVID-19 therapeutics. Peng et al. (2021) constructed a new heterogeneous virus–drug network and developed a novel random walk with restart method (VDA-RWR) for identifying possible virus–drug association related to SARS-CoV-2. The second category is based on deep learning methods. Deepthi et al. (2021) proposed DLEVDA, which inputs features incorporating the pairwise similarities of drug chemical structures and virus genome sequences into a convolutional neural network, and finally infers promising candidates against SARS-CoV-2 infection through an XGBoost classifier. Wang et al. (2021a) proposed the COVIDVS1, COVIDVS-2, and COVIDVS-3. These three models base on a directed message passing neural network. COVIDVS1 first constructed a broad-spectrum anti-beta-coronavirus compound prediction model, and then COVIDVS-2 and COVIDVS-3 were obtained by applying transfer learning to fine-tune the model using the specific anti-SARS-CoV-2 compound datasets.

Although the prior studies have led to significant progress, there is still room for improvement.

- Researchers have been exploring new potential drugs by testing their antiviral activity against SARS-CoV-2 (Zaliani et al. 2022); therefore, the accurate prediction of COVID-19 drug response is of great significance for guiding the design of SARS-CoV-2 antiviral drugs and repurposing existing drugs in the treatment of SARS-CoV-2. However, previous methods are not able to predict COVID-19 drug response.
- The conventional drug feature lacks complete drug chemistry information. For example, the fingerprint-based drug features and similarity network features are insufficient in representing the structure information of drug molecules.
- Pharmacogenomics (Daly 2017) is a subject of exploring the influence of genetic variation of genes on drug treatment effects from the perspective of the genome. For example, transcriptome profiling provides an opportunity to predict antiviral drug response. However, the pharmacogenomics profiles have yet to be well utilized in the study of anti-SARS-CoV-2 drug prediction.
- Due to the limited and sparse publicly available SARS-CoV-2 data, it is challenging to apply deep learning methods to improve the predictive performance of drug response.

In this study, we propose a transfer learning method with a graph transformer for predicting COVID-19 drug response, namely DeepCoVDR. First, we construct a graph transformer that can simultaneously capture the information of neighbor nodes and long-distance node information in drug molecules to extract drug information. Second, we use a feed-forward neural network (DNN) to learn the representation of transcriptomics. Third, a cross-attention module is employed to produce new embeddings of drugs and cell lines which can supplement the relationship between each node in the drug molecule and the corresponding cell line. To solve the problem that the amount of COVID-19 drug response data is very limited, we use cancer cell lines, drugs, and their responses to pretrained our model and then use Vero E6, which is highly permissive to SARS-CoV-2 infection, drugs and their responses to fine-tune the model. DeepCoVDR can not only predict the half-maximal inhibitory concentration (IC50) sensitivity value in the regression task but also classify a drug as sensitive or resistant in the classification task. We have designed many experiments to test the performance of DeepCoVDR. In the classification and regression settings, our model achieves state-of-the-art performance. We also apply our model to screen the FDA-approved drugs from Skuta et al. (2017), and the drug candidates also confirm the accuracy of DeepCoVDR.

## 2 Materials

Deep learning methods usually utilize a large amount of data that provide a good basis for solving various problems. Unfortunately, applying deep learning to predict COVID-19 drug response is a major challenge. SARS-CoV-2, as a newly discovered virus, has very limited data available. This will significantly affect the performance of a model in the potential data space. According to the principle of similarity (Willett 2014; Kubinyi 1998), similar molecule structures may work on the same genes. That shows the feasibility of adopting transfer learning on this issue. A sufficient amount of data helps the model to learn enough knowledge of drugs and cell line during pretraining, so we choose cancer dataset which is large enough for pretraining and easy to collect.

For the cancer dataset, we extract the dataset from Jiang et al. (2022). This dataset mainly uses the new version of the public database GDSC (Yang et al. 2013), GDSC2, containing 135, 242 IC50 values (natural log-transformed) across 809 cell lines and 198 compounds. It selects 17 777 gene expression values from GDSC2 for each cell line and gets the SMILES strings of all compounds from PubChem (Kim et al. 2019).

For the SARS-CoV-2 dataset, we collected a set of IC50 values of 318 drugs on Vero E6 from Zaliani et al. (2022). For the omics data of Vero E6, we use the data from Riva et al. (2020), which contain 16 122 gene expression values. To ensure the consistency of feature dimension, we filter the genes that are different from the cancer dataset. Finally, only 11 793 gene expression values in both the cancer dataset and the SARS-CoV-2 dataset are retained.

## 3 Methods

### 3.1 Overview of DeepCoVDR

**Figure 1.**
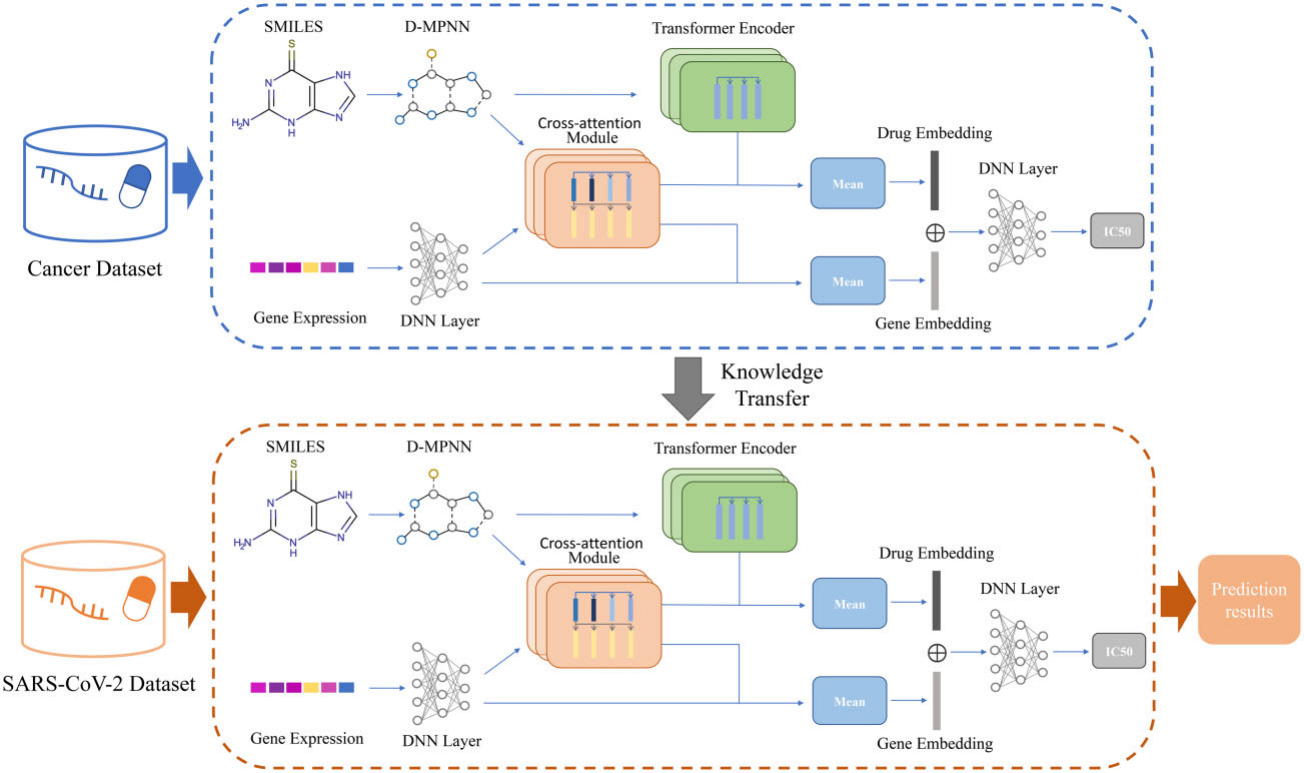
Flowchart of DeepCoVDR. We first use the cancer dataset to pre-train the model and then fine-tune the model on the SARS-CoV-2 dataset. The model consists of three parts: feature representation extraction, cross-attention module, and drug response prediction. The drug’s chemical structure as a graph is input into a graph transformer-based network and transformed into a high-level representation. And the gene expression data is used to extract cell line feature representation by a feed-forward neural network. Then, we employ a cross-attention module to calculate the interaction between the drug and cell line and obtain the representation of fusion information. In drug response prediction, we generate the final embeddings of the drug and cell line and take them as input to predict drug response.

DeepCoVDR is a deep learning method based on graph transformer, cross-attention, and transfer learning for COVID-19 drug response prediction. The flowchart of DeepCoVDR is shown in Fig. 1. DeepCoVDR has three parts: features mining, cross-attention module, and drug response prediction.

In the first part, we extract drug representation and cell line representation from the molecular structure of drugs and gene expression values. For the drug, we first use D-MPNN (Yang et al. 2019) to aggregate information from neighboring atoms and bonds, and then the node embeddings are fed into a transformer encoder to obtain the final representation of the drug. For the cell line, we get the representation through a five-layer DNN network.

In the cross-attention module, we stack the drug node feature from D-MPNN and the broadcasting cell line feature. Under the attention mechanism of the transformer, we get another drug representation and another cell line representation. Both of them contain fusion information.

Finally, we average two drug and two cell line representations separately and concatenate result features. After extraction of a three-layer DNN, we can obtain the predicted result. We take mean square error as the loss function for the regression task. For the classification task, we add an additional sigmoid layer and use the cross-entropy as the loss function. The optimizer in the back-propagation process is Adam. To reduce the impact of data scarcity on model performance, we adopt transfer learning in the model. Transfer learning can address data scarcity by leveraging existing knowledge from source tasks to low-data target tasks (Pan and Yang 2010). We first use the cancer dataset to pretrain the model. Then we fine-tune the pretrained model with the SARS-CoV-2 dataset and obtain the target model.

### 3.2 Drug information extraction

Drugs can be compiled into molecular graphs. Using the open-source package RDKit (https://rdkit.org/docs/index.html), the SMILES string of each drug can be converted into a chemical structure with atom features and bond features. To fully use drug structure information, we construct a graph transformer framework that not only uses D-MPNN (Yang et al. 2019) to learn the local representation of the immediate adjacent structure of nodes but also uses transformer encoder (Vaswani et al. 2017) as the global inference module. As an extension of MPNN (Gilmer et al. 2017), D-MPNN uses messages associated with a directed edge to make use of key attributes and avoid unnecessary loops in the message delivery path (Yang et al. 2019). Taking a drug *D* of *n* atoms as an example, we first initialized edge hidden states: where $W^{i}\in R^{h_{c}\times h}$ is a trainable parameter, $n_{a}$ is atom features for atom a and $e_{ab}$ is bond features for bond *ab*, and *f* is the ReLU activation function (Nair and Hinton 2010). Then, the message-passing process includes *t_f_* steps. The messages and the hidden states of step *t *+* *1 are respectively $m_{ab}^{t+1}$ and $h_{ab}^{t+1}$: where *N_a_* is the set of neighbors of *a* in graph *D*, $W^{m}\in R^{h\times h}$ is a trainable parameter. And the final messages and representation of the atom *a* are $m_{a}$ and $h_{a}$: where $W^{o}\in R^{h\times h}$ is a trainable parameter.


(1) $$h_{ab}^{0}=f\left(\left[n_{a},e_{ab}\right]W^{i}\right)$$

(2) $$m_{ab}^{t+1}=\sum_{i\in \{N_{a}\backslash b\}}h_{ia}^{t}$$

(3) $$h_{ab}^{t+1}=f\left(h_{ab}^{0}+m_{ab}^{t+1}W^{m}\right)$$

(4) $$m_{a}=\sum_{i\in N_{a}}h_{ai}^{t_{f}}$$

(5) $$h_{a}=f\left(\left[n_{a},m_{a}\right]W^{o}\right)$$

Transformer is a deep learning method with attention mechanism for seq2seq tasks (Vaswani et al. 2017). By using a self-attention module, the transformer can extract syntax information effectively. Herein, we use a transformer encoder to mine pairwise message between atom encodings of drug molecules. After we obtain the final pernode encoding $h_{d}\in R^{n\times h}$ of drug *D* from D-MPNN, we input them into a transformer encoder. Transformer mainly includes two parts, a multiattention layer and a feed-forward network. A multiattention layer consists of several self-attention layers which takes the keys, the queries and the values as input to calculate scaled dot-product attention. We first get query $Q_{d}$, key $K_{d}$, and value $V_{d}$ by where $W_{d}^{Q},W_{d}^{K},W_{d}^{V}\in R^{h\times z}$ are learnable weight matrixes. To allow model consider different subspaces, we run *u* parallel self-attention layers and concatenate the output per-head encodings. where $W^{T}\in R^{\text{uz}\times z}$ is a weight parameter and $\frac{1}{\sqrt{z}}$ is a scaled factor. To further adjust the representation, the output of the multiattention layer is input into the feed-forward layer: where $E_{1}^{d}$ is the first drug representation, $W_{1}^{d}\in R^{z\times o},W_{2}^{d}\in R^{o\times o}$, and $b_{d}^{1},b_{d}^{2}\in R^{o}$ are weight parameters.


(6) $$Q_{d}=h_{d}W_{d}^{Q},K_{d}=h_{d}W_{d}^{K},V_{d}=h_{d}W_{d}^{V}$$

(7) $$\begin{array}{cc} {\;\text{head}\;}_{i} & =\begin{array}{c} \text{Attention}(Q_{d},K_{d},V_{d}) \end{array}\\ & =\begin{array}{c} \text{softmax}\left(\frac{Q_{d}K_{d}^{T}}{\sqrt{z}}\right)V_{d} \end{array} \end{array}$$

(8) $$\;\text{MultiHead}\;(h_{d})=\text{concat}\left({\;\text{head}}_{1},\ldots ,{\;\text{head}}_{u}\right)W^{T}$$

(9) $$E_{1}^{d}=f\left(\;\text{MultiHead}\;(h_{d})W_{1}^{d}+b_{d}^{1}\right)W_{2}^{d}+b_{d}^{2}$$

### 3.3 Cell line information extraction

For cell lines, we design a five-layer DNN to extract feature representation from gene expression data. Taking cell line *C* as an example, the first representation of Cell line $E_{1}^{c}$ is the final output of DNN. where i∈[1,5] is the number of layers,$h_{i}^{c}$ is the output vector of layer *i*, $W_{i}^{c}\in R^{d_{i-1}\times d_{i}}$ is weight parameter.


(10) $$h_{i}^{c}=f\left(h_{i-1}^{c}W_{i}^{c}+b_{i}^{c}\right)$$

### 3.4 Cross-attention module

Although we have obtained drug feature representation $E_{1}^{d}$ and cell line feature representation $E_{1}^{c}$, which contain the information of their own characteristics, their mutual connection is ignored. Inspired by multimodality cross attention network for image and sentence matching (Wei et al. 2020), we present a cross-attention module based on a transformer encoder that allows us to calculate the effect of each atom in the molecule and the corresponding cell line, to generate $E_{2}^{d}$ and $E_{2}^{c}$ which are the second representation of drug and cell line containing fusion information. The core operations are as follows. We first use stacked features of drug and cell line to obtain the query $Q_{F}$, key $K_{F}$ and value $V_{F}$: where $W_{f}^{Q},W_{f}^{K},W_{f}^{V}\in R^{h\times f}$ are learnable weight matrices and $h_{c}\in R^{n\times h}$ is obtain from $E_{1}^{c}$ by broadcasting. Then the output of head *i* is calculated as follows: where *s* represent the softmax and scaled function, $E_{2}^{d_{i}}$ and $E_{2}^{c_{i}}$ are the hidden representation of drug and cell line. From $E_{2}^{d_{i}}=s(Q_{D}K_{D}^{T})V_{D}+s(Q_{D}K_{C}^{T})V_{C}$ and $E_{2}^{c_{i}}=s(Q_{C}K_{C}^{T})V_{C}+s(Q_{C}K_{D}^{T})V_{D}$, the cross-attention module is able to consider the interaction of drug feature and cell line feature for the representation of drug and cell line. Similar to formulas 9 and 10, we can get the output of the transformer encoder based on multiattention module ${\left(E_{2}^{d}E_{2}^{c}\right)}^{T}$. $E_{2}^{d}$ and $E_{2}^{c}$ are the second representation of drug and cell line.


(11) $$Q_{F}=\left(\begin{array}{c} Q_{D}\\ Q_{C} \end{array}\right)=\left(\begin{array}{c} h_{d}W_{f}^{Q}\\ h_{c}W_{f}^{Q} \end{array}\right)$$

(12) $$K_{F}=\left(\begin{array}{c} K_{D}\\ K_{C} \end{array}\right)=\left(\begin{array}{c} h_{d}W_{f}^{K}\\ h_{c}W_{f}^{K} \end{array}\right)$$

(13) $$V_{F}=\left(\begin{array}{c} V_{D}\\ V_{C} \end{array}\right)=\left(\begin{array}{c} h_{d}W_{f}^{V}\\ h_{c}W_{f}^{V} \end{array}\right)$$

(14) $$\begin{array}{cc} {\;\text{head}\;}_{i}^{F} & =\begin{array}{c} \text{Attention}(Q_{F},K_{F},Q_{F}) \end{array}\\ & =s\left(\left(\begin{array}{c} Q_{D}\\ Q_{C} \end{array}\right)\left(\begin{array}{cc} K_{D}^{T} & K_{C}^{T} \end{array}\right)\right)\left(\begin{array}{c} V_{D}\\ V_{C} \end{array}\right)\\ & =\left(\begin{array}{cc} s(Q_{D}K_{D}^{T}) & s(Q_{D}K_{C}^{T})\\ s(Q_{C}K_{D}^{T}) & s(Q_{C}K_{C}^{T}) \end{array}\right)\left(\begin{array}{c} V_{D}\\ V_{C} \end{array}\right)\\ & =\left(\begin{array}{c} s(Q_{D}K_{D}^{T})V_{D}+s(Q_{D}K_{C}^{T})V_{C}\\ s(Q_{C}K_{C}^{T})V_{C}+s(Q_{C}K_{D}^{T})V_{D} \end{array}\right)\\ & =\left(\begin{array}{c} E_{2}^{d_{i}}\\ E_{2}^{c_{i}} \end{array}\right) \end{array}$$

### 3.5 Baseline methods

To test the performance of DeepCoVDR, we compared our methods with six baselines: support vector machine (SVM) (Hearst et al. 1998), a random forest (RF) (Breiman 2001), XGBoost (Chen and Guestrin 2016), feed-forward neural network (DNN), graph convolutional network (GCN) Kipf and Welling (2016), and graph attention network (GAT) (Veličković et al. 2017). The input feature of SVM, RF, XGBoost, and DNN are the concatenation of Morgan fingerprints (Morgan 1965) and gene expression data. GCN and GAT can process graph structure data as graph neural network methods, so their inputs are molecular structures that contain atom features and bond features and gene expression data. The two methods use the same way as DeepCoVDR to process gene expression data. Their main difference lies in the treatment of drug structure. The parameters of these baselines are default or the best. The details are as follows. For SVM, RF and XGBoost, we choose the default hyperparameters from sklearn library (Pedregosa et al. 2011). And the hyperparameters of DNN, GCN and GAT are selected by experimental results. For DNN, we set three hidden layers and the dimension of hidden layers are [1024, 512, 128]. For GCN, we use three GCN layers and the dimension are [78, 156, 312]. For GAT, we set six attention heads and two GAT layers which dimension are [128, 128]. The optimizer of DNN, GCN and GAT is Adam.

## 4 Results

### 4.1 Experimental settings and model evaluation

We conduct five 5-fold cross-validations by five different random seeds to evaluate the performance of DeepCoVDR and the competitive methods. The final experimental results come from the average of five 5-fold cross-validations.

For the model evaluation of regression experiment, we use three metrics: Pearson’s correlation coefficient (PCC) (Benesty et al. 2009), Spearman’s correlation coefficient (SCC) (Myers and Sirois 2004) and root mean squared error (RMSE). PCC is a statistic reflecting the linear correlation degree of observed and predicted logarithm IC50. SCC is a nonparametric measure of ranked values and uses a monotone function to describe the relationship between two variables observed and predicted logarithm IC50. And the RMSE is a traditional measure to calculate the level of accuracy.

For the model evaluation of the classification experiment, we use four common metrics: the area under the receiver operating characteristic curve (AUC), the area under the precision-recall curve (AUPR), accuracy (ACC), and F1 score. The F1 score is a harmonic average of accuracy and recall.

### 4.2 Performance comparison on regression experiments

**Figure 2.**
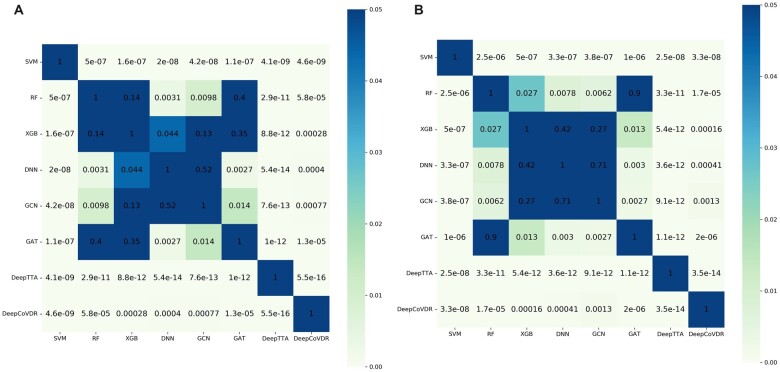
Heat maps of *P*-value obtained by performing *t*-test on the results of regression experiment. (a) is for Pearson’s correlation and (B) is for Spearman’s correlation.

The performance comparison of DeepCoVDR on regression experiment is shown in Table 1. DeepTTA is the state-of-art transformer-based method to predict cancer drug response accurately. To further prove the high performance of DeepCoVDR, we train DeepTTA on our SARS-CoV-2 dataset and add it to the comparison on the regression experiment. From the comparison results, we can have the following analysis: (i) Among the eight methods, DeepCoVDR outperforms other methods, including DeepTTA, by achieving Pearson’s correlation of 0.942, Spearman’s correlation of 0.881, and RMSE of 0.509. Fig. 2 shows the *P*-values of PCC and SCC between each method and other methods. The *P*-value between DeepCoVDR and other methods is less than .05, which proves the credibility of our experimental conclusion and that the improvements of DeepCoVDR have statistical significance; (ii) The methods based on deep learning, including DNN, GCN, GAT, DeepTTA, and DeepCoVDR, perform better than the methods based on traditional machine learning. That shows deep learning has the ability to discover complex information in high-dimensional data. (iii) The methods that only use fingerprints as a drug feature perform worse than the methods that use chemical structures. That shows chemical structures contain more detailed and higher-level drug information.

**Table 1.** Performance comparison of DeepCoVDR and baseline methods on regression experiments.

Methods	PCC	SCC	RMSE
SVM	0.470 ± 0.039	0.438 ± 0.045	1.616 ± 0.008
RF	0.812 ± 0.036	0.730 ± 0.033	0.921 ± 0.061
XGB	0.847 ± 0.032	0.783 ± 0.028	0.826 ± 0.087
DNN	0.887 ± 0.018	0.798 ± 0.027	0.843 ± 0.040
GCN	0.878 ± 0.025	0.805 ± 0.031	0.626 ± 0.063
GAT	0.829 ± 0.024	0.733 ± 0.022	0.926 ± 0.219
DeepTTA	0.910 ± 0.018	0.858 ± 0.024	0.588 ± 0.075
DeepCoVDR	**0.942 ± 0.011**	**0.881 ± 0.017**	**0.509 ± 0.031**

For runtime analysis, we benchmarked DeepCoVDR and DeepTTA on the same server with a single RTX3090 GPU. The training costs of DeepCoVDR and DeepTTA are 3.40 h and 1.72 h, respectively. It is reasonable that DeepCoVDR requires more training time because it utilizes a more complex deep transfer learning approach to improve regression performance.

### 4.3 Performance comparison on classification experiments

**Figure 3.**
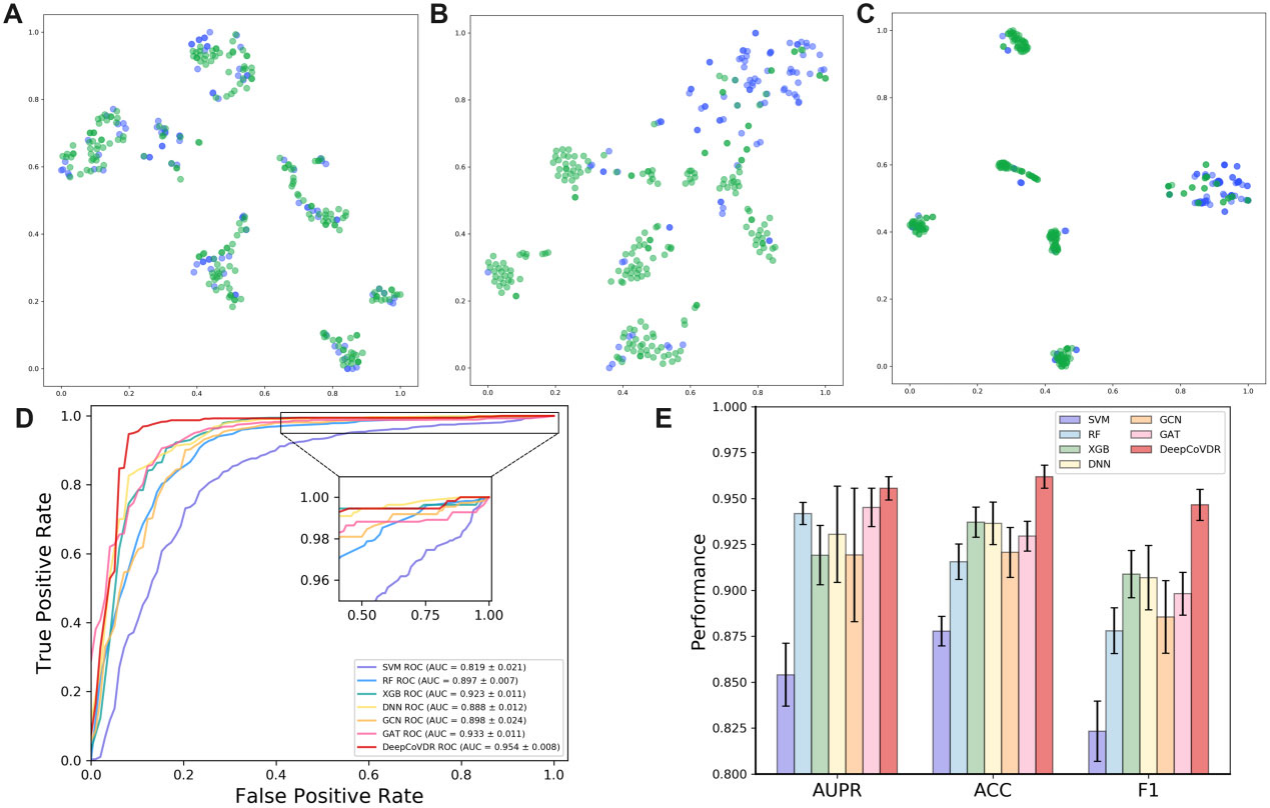
Performance of DeepCoVDR in the classification setting. (a–c ) are visualization of the final embeddings of DeepCoVDR, and they are from epochs 20, 100, and 1000 respectively. The green and blue dots respectively represent the drugs with and without antiviral activity in our dataset. (d) shows the receiver operating characteristic (ROC) curve of the seven comparing methods. (e) shows the AUPR, ACC, and F1 scores of DeepCoVDR and baseline methods.

In this study, we binarize the IC50 values through the threshold 20 *μM* (Zaliani et al. 2022). In order to prove the classification performance of our model, we use t-SNE (Van der Maaten and Hinton 2008) to visualize the classification results of DeepCoVDR. As shown in Fig. 3a–c, the green and blue dots respectively represent the drugs with and without antiviral activity in our dataset. It can be clearly seen that with the increase in the number of training epochs, the dots representing different types of samples are gradually distinguished, and the dots of the same type are gradually gathered. This represents that our model can extract and distinguish hidden knowledge in features. Then, we use AUC, AUPR, ACC, and F1 scores to measure the model’s performance. Figure 3d shows the ROC curves of DeepCoVDR and other baseline methods. More results are shown in Fig. 3e. DeepCoVDR outperforms other baseline methods by achieving significantly higher AUC, AUPR, ACC, and F1 scores of 0.954, 0.956, 0.962, and 0.946. From the above results, we can see that DeepCoVDR has reached the state-of-art of all comparison methods, demonstrating the superiority of DeepCoVDR when performing classification tasks.

### 4.4 Drug response prediction on the cancer dataset

Before we use the SARS-CoV-2 dataset to predict the drug response, we first train DeepCoVDR on the cancer dataset. To demonstrate the high performance of DeepCoVDR, we compare the performance of our model with some advanced cancer drug response models on the cancer dataset. As shown in Table 2, DeepCoVDR achieves state-of-the-art performance on PCC and SCC among the existing models where DeepTTA is the state-of-art transformer-based method. Specifically, the highest PCC 0.946 and SCC 0.935 proves a strong agreement between observed and predicted IC50, and the high performance on the cancer dataset shows our model has strong generalization ability.

**Table 2.** Performance comparison of DeepCoVDR and advanced methods on cancer dataset.

Methods	PCC	SCC
MOLI (Sharifi-Noghabi et al. 2019)	0.813 ± 0.007	0.782 ± 0.005
CDRscan (Chang et al. 2018)	0.871 ± 0.004	0.852 ± 0.003
tCNNs (Liu et al. 2019)	0.910 ± 0.009	0.889 ± 0.008
DeepCDR (Liu et al. 2020)	0.923 ± 0.005	0.898 ± 0.008
DeepTTA	0.941 ± 0.003	0.914 ± 0.004
DeepCoVDR	**0.946 ± 0.001**	**0.935 ± 0.005**

Note: The results of the comparison method are from DeepTTA.

### 4.5 Performance comparison on drug repurposing

**Figure 4.**
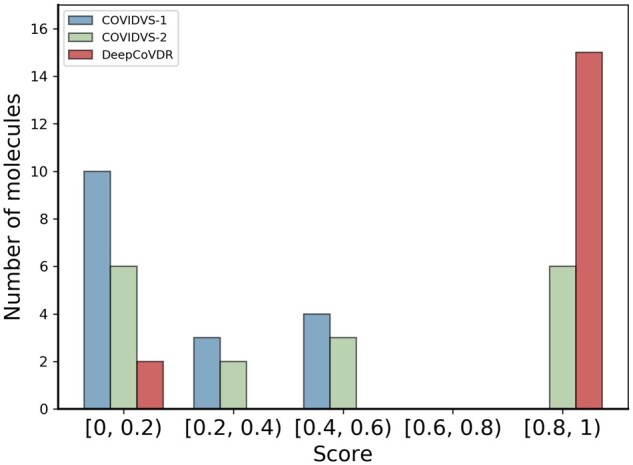
Distribution of scores for 17 ReFRAME actives predicted with COVIDVS-1, COVIDVS-2, and DeepCoVDR. The results of COVIDVS-1, COVIDVS-2 are from Wang et al. (2021a).

To test DeepCoVDR’s ability of repurposing, we compare DeepCoVDR with COVIDVS-1 and COVIDVS-2 on the same ReFRAME actives dataset (Wang et al. 2021a) which contains 17 identified ReFRAME (Riva et al. 2020) actives. There is no overlap between the ReFRAME actives dataset and our SARS-CoV-2 dataset. The result is shown in Fig. 4. Among 17 compounds, 15 got predicted scores more than 0.8 by DeepCoVDR, approximately 82% of all ReFRAME actives. While 6 got predicted scores greater than 0.8 by COVIDVS-2, and no compounds got predicted scores greater than 0.8 by COVIDVS-1. The results demonstrate that our method has a strong ability to screen anti-SARS-CoV-2 drugs.

### 4.6 Ablation study

To further investigate the importance of components, transfer learning, graph transformer framework, gene expression, and the cross-attention module, we design the following variants of DeepCoVDR:

- **NoTransfer** remove transfer learning.
- **NoGraphTransformer** use transformer instead of graph transformer.
- **NoGene** remove the gene expression feature.
- **NoCrossAttention** remove cross-attention module.

The results are shown in Table 3. The experiment on NoTransfer shows that transfer learning can successfully transfer the knowledge of drugs and gene expression values from cancer to predict COVID-19 drug response. The performance of NoGraphTransformer shows the importance of graph transformers in drug information mining. When we remove gene expression values, the degradation of model performance indicates the information on the cell line is useful for prediction. And the experiment on NoCrossAttention demonstrates the cross-attention module is effective. The training time of NoTransfer, NoGraphTransformer, NoGene, and NoCrossAttention is 3.24 h, 1.31 h, 2.79 h, and 2.29 h, respectively. Although the training time of DeepCoVDR (3.40 h) is slightly longer, the performance is significantly improved.

**Table 3.** Ablation study.

Methods	PCC	SCC
NoTransfer	0.910 (↓ 3.40%)	0.873 (↓ 0.91%)
NoGrapghTransformer	0.912 (↓ 3.18%)	0.861 (↓ 2.27%)
NoGene	0.922 (↓ 2.12%)	0.853 (↓ 3.18%)
NoCrossAttention	0.925 (↓ 1.80%)	0.853 (↓ 3.18%)
DeepCoVDR	**0.942**	**0.881**

Note: The content in parentheses shows the performance delta percentage of each variant of DeepCoVDR.

### 4.7 Screening FDA-approved drugs to identify novel anti-SARS-CoV-2 drugs

Finding potential targets from existing drugs is still an important way of finding a drug that can treat SARS-CoV-2. We applied DeepCoVDR to screen the 1871 FDA-approved drugs from (Skuta et al. 2017). We removed the drugs that are present in our SARS-CoV-2 dataset, and finally, 1853 drugs are left. To improve the performance, we integrated five models through ensemble technique (Dietterich 2000) technology and used the ensemble model for screening. To verify the reliability of the prediction results of DeepCoVDR, we conducted a nonexhaustive quality search on the top 100 compounds and found that at least 11 compounds have been proven effective against SARS-CoV-2 in previous studies (Table 4).

**Table 4.** Drugs predicted by DeepCoVDR in FDA-approved drugs with the support of literature.

Compound name	Status	Ranking position	PMID
Orlistat	Preclinical	6	34580494
Ritonavir	Phase 3	8	33243253
Artesunate	Preclinical	17	34272426
Folic acid	Phase 3	23	36060767
Artemether	Preclinical	28	34527599
Valproic acid	Phase 4	31	34635175
Adefovir dipivoxil	Preclinical	34	34440200
Remdesivir	Approved (FDA)	41	32366720
Lopinavir	Phase 4	61	33290767
Diltiazem	Preclinical	76	35176124
Bortezomib	Preclinical	96	36335206

We select the following examples for detailed descriptions. Orlistat, a US FDA-approved drug, is used for the treatment of obesity by preventing the absorption of dietary fat and fatty acid synthase to reduce lipid synthesis (Kridel et al. 2004), which also significantly inhibits the infectivity of the SARS-CoV-2 virus with an EC50 of 0.39 μ*M* in vitro (Chu et al. 2021). Remdesivir is a broad-spectrum antiviral compound and was approved for the treatment of COVID-19 in 2020. With strong and stable anti-SARS-CoV-2 activity, remdesivir was used as a reference drug in several Vitro studies with IC50 of ∼10μM (Jeon et al. 2020; Jang et al. 2021). Lopinavir, ranked at position 61, is an antiretroviral protease inhibitor against HIV infections, and its antiviral effects on SARS-CoV-2 are found to have IC50 of 13.16 μM in vitro (Raj et al. 2021). Ritonavir, ranked at position 8, is an HIV-1 protease inhibitor and CYP3A inhibitor used in combination with other antivirals to treat HIV infection and shows some ability in the inhibition of SARS-CoV-2 *M^pro^* with IC50 of 13.7 nM (Mahdi et al. 2020). Notably, Ritonavir may be regarded as a fixed-dose combination product with other drugs to treat COVID-19, such as lopinavir or nirmatrelvir. Nirmatrelvir/ritonavir, commercially named Paxlovid, was granted emergency use authorization by the US FDA for the treatment of COVID-19 in December 2021. Recent research shows that symptomatic COVID-19 patients treated with nirmatrelvir plus ritonavir, have a lower risk of progression to severe COVID-19 compared with placebo (Hammond et al. 2022). However, despite the antiviral effects on SARS-CoV-2 of lopinavir and ritonavir in vitro, a randomized trial found lopinavir/ritonavir was not effective in treating COVID-19 patients in 2020 (Cao et al. 2020). Therefore, more effort is still needed to discover other effective antiviral medications. And above all, our model can identify certain potential candidates with significant antiviral activity in vitro.

## 5 Conclusion and discussion

In this article, we propose a novel deep learning model to predict COVID-19 drug response based on graph transformer, cross-attention, and transfer learning. To the best of our knowledge, DeepCoVDR is the first computing method that can accurately predict COVID-19 drug response value, and it is also the first work to apply graph transformer and cross-attention in a drug response prediction tasks. Our model achieves state-of-art performance in all metrics, according to the comparison experiment results of regression and classification tasks, compared with baseline methods. DeepCoVDR also achieves high performance in cancer drug response prediction, showing its high versatility. In addition, we demonstrate DeepCoVDR can repurpose existing drugs for the treatment of SARS-CoV-2 through screening FDA-approved drugs to identify novel anti-SARS-CoV-2 drugs.

In the future, we will explore more multisource heterogeneous information for COVID-19 drug response prediction, e.g. the priority of gene expression value in cell lines that is significant in revealing the potential therapeutic targets of anti-SARS-CoV-2 drugs.
